# Genetic Correlation and Bidirectional Causal Association Between Type 2 Diabetes and Pulmonary Function

**DOI:** 10.3389/fendo.2021.777487

**Published:** 2021-11-25

**Authors:** Jiahao Zhu, Huanling Zhao, Dingwan Chen, Lap Ah Tse, Sanjay Kinra, Yingjun Li

**Affiliations:** ^1^ Department of Epidemiology and Health Statistics, School of Public Health, Hangzhou Medical College, Hangzhou, China; ^2^ JC School of Public Health and Primary Care, The Chinese University of Hong Kong, Hong Kong, Hong Kong SAR, China; ^3^ Department of Non-Communicable Disease Epidemiology, London School of Hygiene & Tropical Medicine, London, United Kingdom

**Keywords:** glycemic traits, linkage disequilibrium score regression, pulmonary function, Mendelian randomization, type 2 diabetes

## Abstract

**Background:**

Observational studies have shown possible bidirectional association between type 2 diabetes (T2D) and pulmonary function, but the causality is not well defined. The purpose of this study is to investigate genetic correlation and causal relationship of T2D and glycemic traits with pulmonary function.

**Methods:**

By leveraging summary statistics from large-scale genome-wide association studies, linkage disequilibrium score regression was first implemented to quantify genetic correlations between T2D, glycemic traits, and several spirometry indices. Then both univariable and multivariable Mendelian randomization analyses along with multiple pleiotropy-robust methods were performed in two directions to assess the causal nature of these relationships.

**Results:**

Forced expiratory volume in 1 s (FEV1) and forced vital capacity (FVC) showed significant genetic correlations with T2D and fasting insulin levels and suggestive genetic correlations with fasting glucose and hemoglobin A1c. In Mendelian randomization analyses, genetically predicted higher FEV1 (OR = 0.77; 95% CI = 0.63, 0.94) and FVC (OR = 0.82; 95% CI = 0.68, 0.99) were significantly associated with lower risk of T2D. Conversely, genetic predisposition to higher risk of T2D exhibited strong association with reduced FEV1 (beta = −0.062; 95% CI = −0.100, −0.024) and FEV1 (beta = −0.088; 95% CI = −0.126, −0.050) and increased FEV1/FVC ratio (beta = 0.045; 95% CI = 0.012, 0.078). We also found a suggestive causal effect of fasting glucose on pulmonary function and of pulmonary function on fasting insulin and proinsulin.

**Conclusions:**

The present study provided supportive evidence for genetic correlation and bidirectional causal association between T2D and pulmonary function. Further studies are warranted to clarify possible mechanisms related to lung dysfunction and T2D, thus offering a new strategy for the management of the two comorbid diseases.

## Introduction

Type 2 diabetes (T2D) affected 425 million people worldwide in 2017, and it is estimated to affect 629 million people by 2045 (1). T2D is associated with the development of devastating microvascular complications, such as nephropathy and impaired renal function, retinopathy and visual loss, and peripheral sensory and autonomic neuropathy (2, 3). Although traditional diabetic complications have not classically included lung diseases and impaired pulmonary function, the lung parenchyma is considered to be a potential target for its great vascularization and abundant collagen and elastin fibers (4).

Accumulating evidence has shown an association between T2D and several respiratory parameters for pulmonary function such as forced expiratory volume in 1 s (FEV1), forced vital capacity (FVC), and the FEV1/FVC ratio (5–8). On the reverse direction, Wannamethee et al. found that FEV1 and FVC were significantly and inversely associated with incident T2D after adjustment for demographic and metabolic factors in a prospective study including 4,434 adults in 24 British towns who were followed up for 20 years (9). However, potential residual confounding such as smoking and obesity as well as the uncertainty of the onset time and delayed diagnosis of the two functional disorders may confer substantial biases in the abovementioned observational studies (10, 11). Thus, the complicated bidirectional association between T2D and pulmonary function needs to be disentangled in a method that can largely avoid the reverse causality and residual confounding.

The Mendelian randomization (MR) is a widely adopted analytic tool that follows the law of independent assortment and uses genetic variants such as instrumental variables (IVs) to investigate the causal effects of exposure on outcome in current medicine (12). Since the genotype of an individual is determined at conception and cannot be changed, this method therefore largely avoids the reverse causality between the genetic phenotype and the associated outcome. Moreover, the causal estimates from MR are minimally affected by effects from residual confounding, as genotype is independent of behavioral or environmental factors.

In the present study, we applied a two-sample bidirectional MR framework to figure out the bidirectional causal associations of pulmonary function parameter, estimated by FEV1, FVC, and FEV1/FVC ratio, with four glycemic traits (fasting glucose, fasting insulin, glycated hemoglobin A1c [HbA1c], and fasting proinsulin levels) as well as risk of T2D.

## Material and Methods

### Study Design

**Figure 1:**
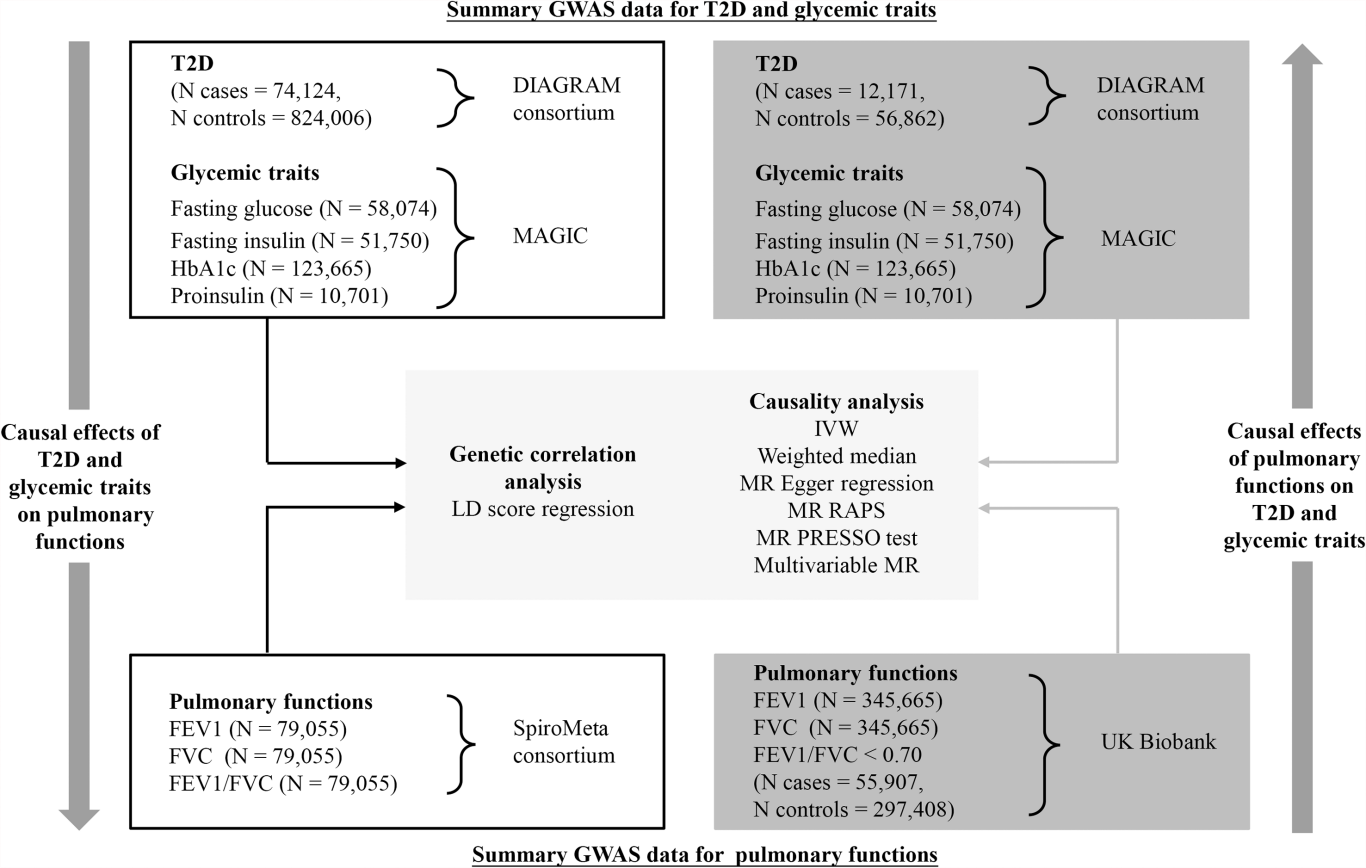
Schematic overview of the study design. FEV1, forced expiratory volume in 1 s; FVC, forced vital capacity; GWAS, genome-wide association study; HbA1c, hemoglobin A1c; MR, Mendelian randomization; T2D, type 2 diabetes.

An overview of the study design is illustrated in **Figure 1**. Briefly, with large-scale summary statistics from independent meta-analyses of genome-wide association study (GWAS), we first performed linkage disequilibrium (LD) score regression to explore genetic correlations of T2D and glycemic traits with pulmonary function. Then, bidirectional two-sample MR analyses with multiple complementary analyses were conducted to unpick the causality/directionality of these associations. In order to select more genetic variants as IVs for T2D and pulmonary function, we utilized different datasets to analyze the two directions. This work was reported in accordance with the STROBE-MR guidance (13). Publicly available datasets generated by published GWAS or consortiums were used for analyses. Therefore, no additional ethical approval would be required.

### Data Sources and Instrumental Variable Selection for Examining Causal Effects of Type 2 Diabetes on Pulmonary Function

Summary statistics for T2D were derived from the hitherto largest GWAS released by the DIAbetes Genetics Replication And Meta-analysis (DIAGRAM) consortium, which combined 32 studies from up to 807,553 participants of European ancestry (74,124 cases and 824,006 controls) (14). The GWAS model had been adjusted for age, sex, ancestry, and study-specific covariates if needed. In addition to T2D, we also examined several glycemic traits for secondary analyses. Summary statistics for fasting glucose (N = 58,074), fasting insulin (N = 51,750), HbA1c (N = 123,665), and fasting proinsulin levels (N = 10,701) were available on Meta-Analyses of Glucose and Insulin-related traits Consortium (MAGIC), which meta-analyzed several GWASs to discover genetic regions associated with glycemic traits (15–17). Only individuals of European ancestry without diabetes were included in the GWAS. Adjustment had been made for age, sex, ancestry, and study-specific covariates, whenever appropriate. Of note, the original GWAS for T2D, fasting glucose, and insulin provided both genetic association data with and without body mass index (BMI) adjustment. To avoid collider bias (18), we only analyzed genetic data that had not been adjusted for BMI.

We selected single-nucleotide polymorphisms (SNPs) associated with T2D or glycemic traits at the genome-wide significance (*p* < 5 × 10^−8^) from the original literature. Significant variants within 500 kb of a lead (most significantly associated) variant were considered as independent SNPs. Palindromic SNPs with mirror allele frequency <0.3 were regarded strand-ambiguous; and therefore, we removed them in the present investigation. To make the IVs satisfy the MR assumptions, SNPs exhibiting significant associations with both T2D/glycemic traits and pulmonary function were excluded from all the analyses after screening each selected SNP and its proxy (r^2^ > 0.8) in PhenoScanner (19) and GWAS catalog.

Corresponding summary statistics for spirometry indices, including FEV1, FVC, and FEV1/FVC ratio, were acquired from a non-overlapping meta-analysis of 22 studies supported by SpiroMeta consortium, involving 79,055 participants of European ancestry (20). The original GWASs were adjusted for age, sex, and height.

### Data Sources and Instrumental Variable Selection for Examining Causal Effects of Pulmonary Function on Type 2 Diabetes

In reverse direction analysis, we retrieved the summary statistics for “best measures” of pre-bronchodilator FEV1 (N = 345,665), FVC (N = 345,665), and FEV1/FVC < 0.70 (Ncase = 55,907, Ncontrol = 297,408) from the UK Biobank in British White of European ancestry, which were generated by the MRC-IEU consortium and a previous MR study on pulmonary function (21). The GWAS model was adjusted for sex and study-specific covariates, where appropriate.

For IV selection strategy, we first extracted SNPs associated with each spirometry index that reached genome-wide significance threshold (*p* < 5 × 10^−8^). Then, these extracted SNPs were pruned using the PLINK clumping algorithm (r^2^ = 0.001 and clumping distance = 10,000 kb) to ensure that they are mutually independent. We further removed SNPs that showed pleiotropic associations with T2D and/or glycemic traits and are strand-ambiguous.

Corresponding summary statistics for T2D were acquired from another GWAS meta-analysis (without UK Biobank participants) released by the DIAGRAM consortium, including 69,033 subjects of European ancestry (12,171 cases and 56,862 controls) (22). Since the GWAS of glycemic traits did not include the UK Biobank population, the abovementioned GWAS for four examined glycemic traits were still used to ascertain causal effects of pulmonary function on glycemic traits. Detailed information on genotyping, imputation, and quality control of GWAS included in this study has been described in the original publications.

### Linkage Disequilibrium Score Regression

To study the extent to which heritability of T2D/glycemic traits and pulmonary function are shared, we conducted LD score regression analysis based on LD Hub (23). Such a method involves regressing summary-level data from millions of genetic variants across the genome on a measure of each variant’s ability to tag other variants locally (its LD score). In contrast to MR, LD score regression usually focuses on the amounts of genetic correlations and could distinguish population stratification and polygenicity in GWAS. All summary-level data were Z-transformed and quality controlled to meet analytical requirements.

### Mendelian Randomization Analyses

We harmonized the SNP-exposure and SNP-outcome data to rule out strand mismatches and ensure the alignment of SNP allele. The Wald ratios were calculated for each SNP and were combined using inverse variance-weighted (IVW) method to derive overall causal estimates (24, 25). Given that IVW assumes balanced pleiotropy and its estimates may be biased in the presence of pleiotropic IVs, several sensitivity analyses including weighted median, MR-Egger regression, MR robust adjusted profile score (MR-RAPS), and MR pleiotropy residual sum and outlier (MR-PRESSO) were performed to further strengthen the causal inference (26–29). In addition, multivariable MR (30) was carried out to adjust for indirect effects of BMI and smoking by using UK Biobank data generated by the MRC-IEU consortium. These two traits were assumed as major confounders in this study. Horizontal pleiotropy was tested according to MR-Egger intercept (*p* < 0.05) (31). Cochran’s Q test was applied to quantify the heterogeneity in IVW estimates (*p* < 0.05). The causal association between two traits was oriented using Steiger’s test (32). We also carried out “leave-one-out” analysis by removing each SNP in turn to evaluate whether the pooled results were driven by a single variant.

The variance (*R* ^2^) in each trait explained by the SNPs was calculated using a previously reported formula (33). The strength of IVs was measured based on *F*-statistic. We estimated the *a priori* statistical power using an online calculator (https://sb452.shinyapps.io/power/).

Statistical analyses were performed using RStudio version 3.6.2 with “TwoSampleMR” and “MRPRESSO” packages. We used the Bonferroni correction (corrected *p*: 0.05/30 groups for bidirectional analyses = 0.002) to penalize multiple testing. A two-sided *p*-value <0.002 was deemed as statistically significant.

## Results

### Linkage Disequilibrium Score Regression

**Figure 2:**
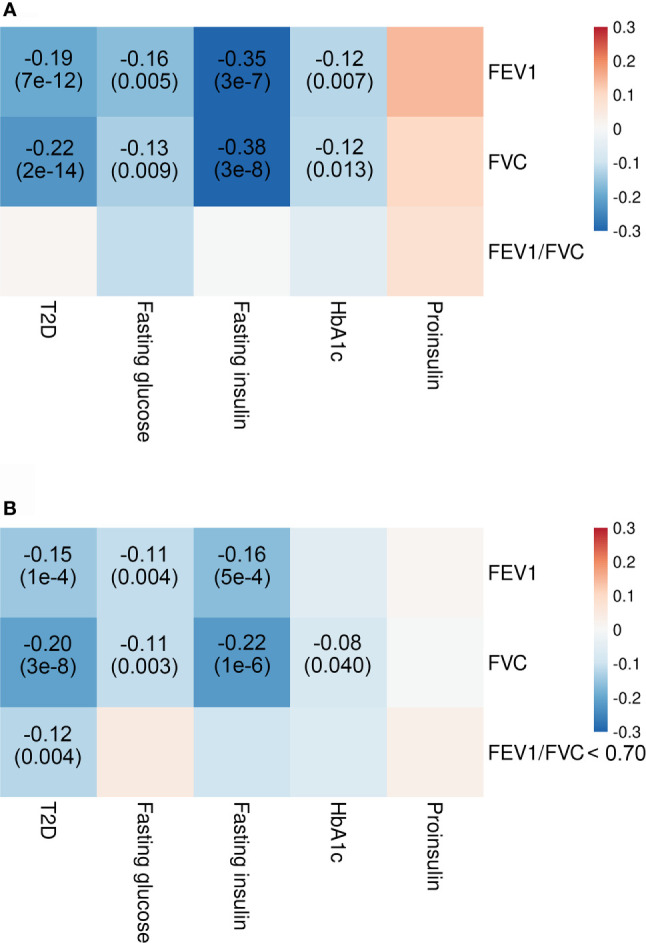
Genetic correlations of T2D and glycemic traits with pulmonary function. Shown are the results from LD score regression with data **(A)** for T2D from DIAGRAM consortium (N = 807,553), for glycemic traits from MAGIC (N = 10,701–123,665), and for pulmonary function from SpiroMeta consortium (N = 79,055) and data **(B)** for T2D from DIAGRAM consortium (N = 69,033), for glycemic traits from MAGIC (N = 10,701–123,665), and for pulmonary function from UK Biobank (N = 345,665–353,315). Colors represent the effect estimates (as measured by r_G_) between two inherited traits, and r_G_ with corresponding *p*-values are only displayed for genetic correlation with *p* < 0.05. Red indicates positive genetic correlation, and blue indicates negative genetic correlation. FEV1, forced expiratory volume in 1 s; FVC, forced vital capacity; HbA1c, hemoglobin A1c; LD, linkage disequilibrium; T2D, type 2 diabetes.

Our analyses showed significant genetic correlations of two spirometry indices, FEV1 and FVC with T2D (FEV1: r_G_ = −0.19; SE = 0.03; *p* = 7e−12; FVC: r_G_ = −0.22; SE = 0.03; *p* = 2e−14) and fasting insulin levels (FEV1: r_G_ = −0.35; SE = 0.07; *p* = 3e−7; FVC: r_G_ = −0.38; SE = 0.07; *p* = 3e−8) after adjusting for multiple testing (**Figure 2**). There was also suggestive evidence supporting genetic correlations of FEV1 and FVC with fasting glucose and HbA1c. Replication analysis using alternative summary statistics yielded a similar pattern of the primary results.

### Causal Effect of Type 2 Diabetes and Glycemic Traits on Pulmonary Function

Through a series of rigorous exclusion criteria, we finally included 192 SNPs for T2D, seven for fasting glucose, six for fasting insulin, 43 for HbA1c, and eight for fasting proinsulin. The *F*-statistics for all traits of interest were much greater than 10, which implied that our MR analyses are unlikely to be susceptible to weak instrument bias. The characteristics of the SNPs used as IVs are shown in **Supplementary Tables 1**–**8**. The information on *a priori* power calculation is presented in **Supplementary Table 9**.

**Figure 3:**
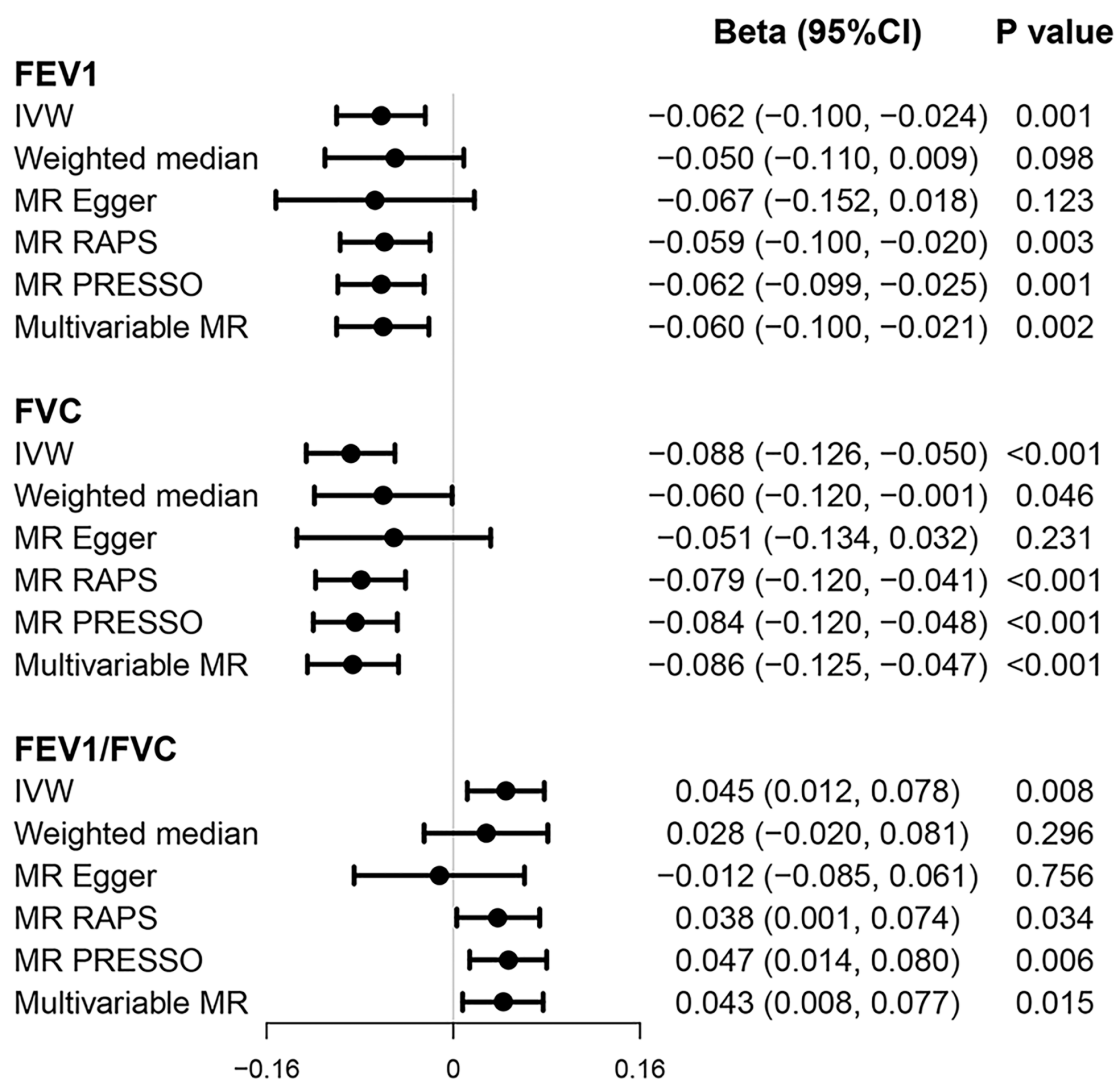
Causal effects of T2D on pulmonary function. Beta (95% CI) represent per one-unit increase in log_e_ odds of T2D. FEV1, forced expiratory volume in 1 s; FVC, forced vital capacity; T2D, type 2 diabetes.

We found a significant association between genetic predisposition to higher risk of T2D and reduced FEV1 (multivariable beta = −0.060; 95% CI = −0.100, −0.021; *p* = 0.002) and FVC (multivariable beta = −0.086; 95% CI = −0.125, −0.047; *p* < 0.001) and increased FEV1/FVC ratio (multivariable beta = 0.043; 95% CI = 0.008, 0.077; *p* = 0.015) in both univariable and multivariable models (**Figure 3**). The results were non-significant when we applied weighted median and MR-Egger methods, which produced causal estimates with wider CIs. However, the direction of point estimates from all established MR models is basically concordant.

Our results also showed some evidence that genetically predicted fasting glucose levels were negatively associated with FEV1/FVC ratio (univariable beta = −0.270; 95% CI = −0.488, −0.053; *p* = 0.015) (**Supplementary Tables 10**–**13**). Similar results were observed in other MR models, with *p*-values less than 0.05 but not exceeding 0.002 after Bonferroni correction. This causal association was not diminished in multivariable MR accounting for BMI and smoking (multivariable beta = −0.273; 95% CI = −0.463, −0.080; *p* = 0.015).

For some traits such as HbA1c, there is some evidence to report the presence of heterogeneity among the SNPs. However, MR-Egger regression demonstrated that our results are unlikely to be affected by pleiotropic effects. Steiger’s test confirmed that the direction of causal association is correct. In the “leave-one-out” analysis, no single SNP was found to significantly affect the results.

### Causal Effect of Pulmonary Function on Type 2 Diabetes and Glycemic Traits

A total of 205 SNPs for FEV1, 264 for FVC, and 106 for FEV1/FVC < 0.70 were identified as valid IVs, which explained 3.38%, 4.76%, and 1.77% of variance, respectively. The *F*-statistics for FEV1, FVC, and FEV1/FVC < 0.70 are 59, 65, and 59, respectively (**Supplementary Tables 6**–**8**). Our study had sufficient power to detect very weak associations of pulmonary function with T2D and glycemic traits (**Supplementary Table 9**).

**Figure 4:**
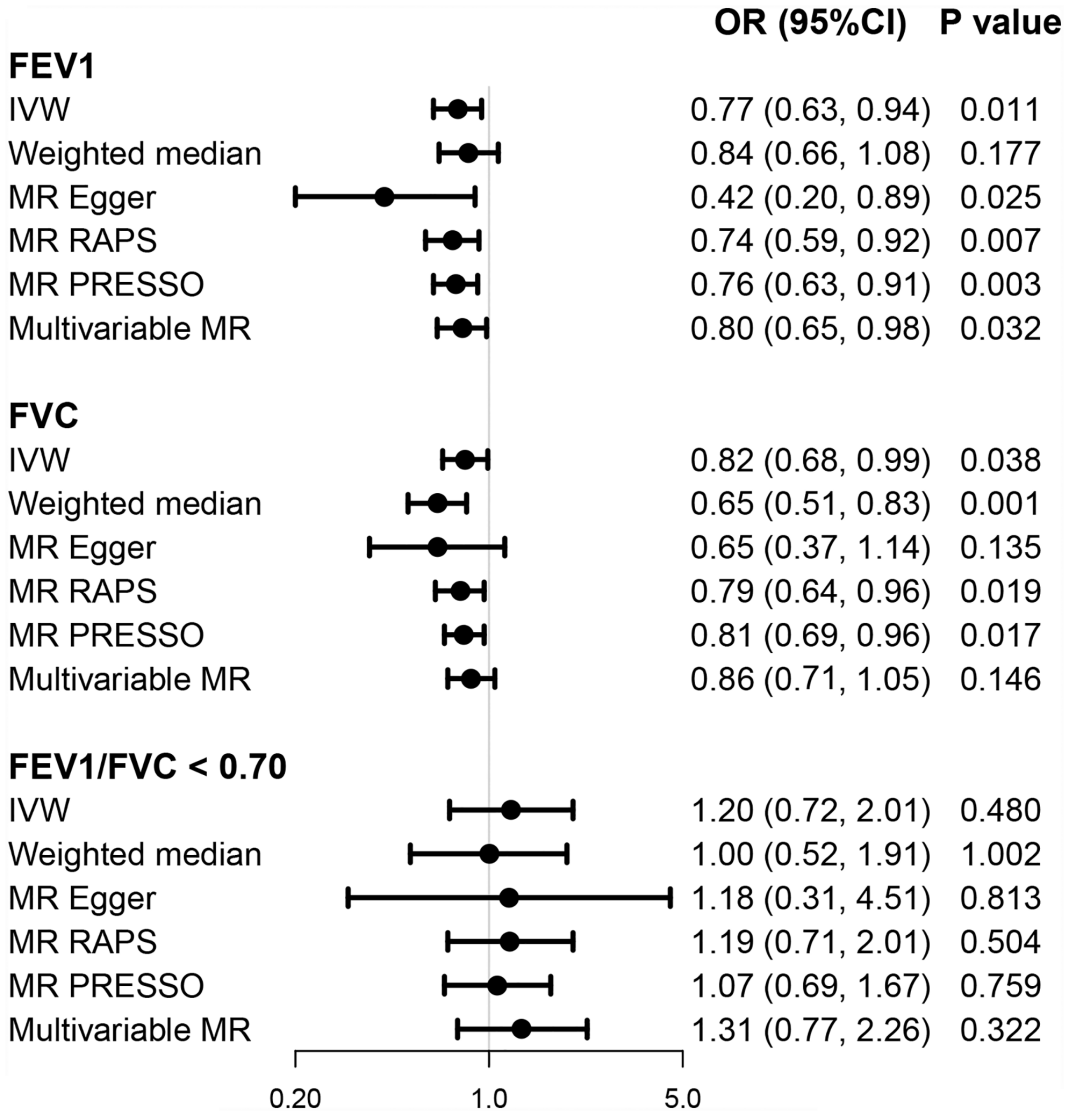
Causal effects of pulmonary function on T2D. OR (95% CI) represents per one-SD increase in FEV1 and FVC and per one-unit increase in log_e_ odds of FEV1/FVC < 0.70. FEV1, forced expiratory volume in 1 s; FVC, forced vital capacity; OR, odds ratio; T2D, type 2 diabetes.

Genetically predicted higher FEV1 (univariable OR = 0.77; 95% CI = 0.63, 0.94; *p* = 0.011) and FVC (univariable OR = 0.82; 95% CI = 0.68, 0.99; *p* = 0.038), but not FEV1/FVC ratio < 0.70 (OR=1.20; 95% CI = 0.72, 2.01; *p* = 0.480), was associated with lower risk of developing T2D based on multiple MR methods (**Figure 4**). Multivariable MR yielded a weaker but still suggestively significant association between genetically predicted FEV1 and the T2D risk after conditioning for BMI and smoking (multivariable OR = 0.80; 95% CI = 0.65, 0.98; *p* = 0.032). However, the causal effect of FVC on T2D was less significant in multivariable analyses (multivariable OR = 0.86; 95% CI = 0.71, 1.05; *p* = 0.146) (**Figure 4**).

We found suggestive evidence for association of genetically predicted FEV1 (multivariable beta = 0.126; 95% CI = 0.030, 0.222; *p* = 0.010) and FVC (beta = −0.095; 95% CI = 0.019, 0.171; *p* = 0.015) with fasting proinsulin levels in most MR models. In addition, there are suggestive associations of genetically predicted FVC (beta = −0.041; 95% CI = −0.077, −0.006; *p* = 0.025) and FVC/FEV1 < 0.70 (beta = 0.419; 95% CI = 0.096, 0.742; *p* = 0.013) with fasting insulin in a single MR model (**Supplementary Tables 14**–**16**).

MR-Egger intercept showed some evidence of possible pleiotropy in the analysis of FEV1/FVC < 0.70 and fasting insulin (*p* = 0.016). The direction of the associations was detected to be corrected by Steiger’s test. “Leave-one-out” analysis suggested that no single SNP has substantial impact on the overall results.

## Discussion

The present study systematically assessed the genetic correlation and causal relationship of T2D and glycemic traits with pulmonary function using large-scale summary GWAS data. LD score regression analyses showed significant and negative genetic correlations of T2D and insulin levels with pulmonary function, indicating a shared genetic basis between these traits. In further MR analyses, we found strong evidence supporting bidirectional causal association between T2D and pulmonary function. There is also a suggestive causal effect of fasting glucose on pulmonary function and of pulmonary function on fasting insulin and proinsulin levels.

A close association of T2D with pulmonary dysfunction has been increasingly reported, but there is still controversy as to which condition occurred first. The majority of studies to date consistently supported that impaired pulmonary function was significant related to the risk of incident T2D. FVC or FEV1 could serve as an independent predictor for T2D progression (11, 34). By contrast, whether T2D can accelerate the decline of pulmonary function is still under debate, with contradictory conclusions drawn from longitudinal data (35, 36). In a meta-analysis combining a total of 93 studies, Zhang et al. documented a possible bidirectional association between T2D and pulmonary dysfunction (37). Of note, a recent MR study examined the causal relationship of T2D and pulmonary function, with only a possible protective effect of higher lung function on T2D being supported, but not the reverse direction (38). By using robust IVs from the largest GWASs for T2D and pulmonary function respectively, our MR investigation provides genetic support for a bidirectional causal association between T2D and pulmonary dysfunction in the general population.

As a well-recognized contributing factor to T2D, insulin resistance (IR) may offer a main pathophysiological explanation for pulmonary abnormalities in T2D (39). Lecube et al. reported that individuals with homeostatic model assessment of IR (HOMA-IR) ≥3.8 were associated with 7.6% decline in FEV1 and 16.4% reduction in maximum midexpiratory flow as compared with those with HOMA-IR < 3.8 in 75 non-diabetic women with no history of cardiovascular or respiratory disease (40). Hyperinsulinemia may promote airway smooth muscle cell proliferation, induce collagen release, and cause a significant increase in calcium response and mitochondrial respiration in cells, leading to adverse effects on the lung tissue (41). In the same case–control study conducted by Lecube et al., the degree of blood glucose control was reported to be associated with respiratory parameters including FEV1/FVC ratio (42). This evidence suggested that hyperglycemia-related metabolic pathways strongly account for lung impairment. Non-enzymatic glycosylation of connective tissue may be a key molecular pathway that induces loss of lung elasticity in patients with T2D (43). Other suggested mechanisms such as low-grade chronic inflammation state, autonomic neuropathy, and lung microangiopathy also seem to play a role in the relation between pulmonary dysfunction and T2D (3). Conversely, there is little knowledge about the mechanisms of lung dysfunction on T2D. A widely accepted hypothesis is that lung-related inflammation may conceivably contribute to the T2D development. Our MR analysis suggested that fasting insulin (surrogate measures of IR) and proinsulin may mediate this association.

In the current study, the possibility of a causal link between pulmonary function and glycemic traits cannot be ruled out, though only an individual MR model computed significant causal estimates, or *p*-value did not reach the corrected threshold. As revealed in LD score regression analyses, FEV1 and FVC were genetically correlated with multiple glycemic parameters, including fasting glucose, HbA1c, and fasting insulin levels. These findings provided support for a causal association between heritable traits. Since two-sample MR is prone to false-negative results, identification of more SNPs that proxy glycemic traits robustly and application of novel genetic correlation methods such as LOGODetect are needed to provide more conclusive results in future studies (44).

Our findings have several crucial implications. On the one hand, FEV1 seems to be a stronger clinical measure (spirometry) to independently predict the incidence of T2D than FVC, as a causal effect of FVC was less significant in multivariable analyses. Clinicians may screen the FEV1 as a convenient and low-cost way in individuals at high risk of T2D. It is also likely to achieve appropriate public health significance on T2D prevention and control through controlling the pulmonary function decline. On the other hand, this study supported a role of T2D in initiating lung abnormalities. Given that the lung may be a new target for T2D-associated microangiopathy, strengthening the management of lung function in T2D patients should be recommended.

There are several limitations to our study. First, genotype is fixed at conception; hence, the MR estimates are interpreted as the impact of lifelong exposure on the outcome, which may differ in the magnitude of the effect size from observational or clinical studies. Second, in the analysis of causal association of T2D and pulmonary function, the GWAS for pulmonary function from SpiroMeta consortium was additionally adjusted for height, making the collider bias possible. Nevertheless, we do not think that this will significantly affect our results, as summary GWAS data for pulmonary function were used as outcome. In this regard, bias from the effect estimate of the SNP associated with pulmonary function is small. Third, the validity of MR results depends on several core assumptions, from which horizontal pleiotropy is considered the major challenge in causal inference. To minimize the impact of horizontal pleiotropy, SNPs exhibiting associations with both T2D/glycemic traits and pulmonary function were manually excluded from all the analyses. We also carried out multiple analytical methods robust to pleiotropy to check the stability of our results. Our findings are broadly consistent across different MR analytical models. Fourth, the use of summary-level data in this MR study means that we were unable to perform stratified analyses by sex, age, overweight status, and smoking. Thus, the potential modifiable role of these characteristics should be further explored in MR study with individual-level data. Finally, the data used for the MR analysis were based on individuals of European descent. While restricting the investigation to racially homogeneous samples can eliminate the population stratification bias, our findings may not be suitable for generalization to other ethnic and cultural groups.

In summary, we found negative genetic correlations of T2D and some glycemic parameters with pulmonary function. Further analyses provided strong evidence supporting bidirectional causal association between T2D and pulmonary function in the general population. Suggestive causal effects of fasting glucose on pulmonary function and of pulmonary function on fasting insulin and proinsulin were also revealed. Further studies are warranted to clarify possible mechanisms related to lung dysfunction and T2D, thus offering a new strategy for the management of the two comorbid diseases.

## Data Availability Statement

The summary statistics analyzed in the study are included in the article/**Supplementary Material**. Further inquiries can be directed to the corresponding author.

## Author Contributions

JZ and HZ wrote the manuscript. DC, LT, SK, and YL interpreted the data and reviewed the manuscript. JZ and HZ designed the study and analyzed the data. YL is the guarantor of this work and, as such, had full access to all the data in the study and takes responsibility for the integrity of the data and the accuracy of the data analysis. All authors contributed to the article and approved the submitted version.

## Conflict of Interest

The authors declare that the research was conducted in the absence of any commercial or financial relationships that could be construed as a potential conflict of interest.

## Publisher’s Note

All claims expressed in this article are solely those of the authors and do not necessarily represent those of their affiliated organizations, or those of the publisher, the editors and the reviewers. Any product that may be evaluated in this article, or claim that may be made by its manufacturer, is not guaranteed or endorsed by the publisher.
